# Living Kidney Transplantation in a Patient With End‐Stage Kidney Disease Due to Rare Juvenile Nail‐Patella‐Like Renal Disease Without *LMX1B* Mutation

**DOI:** 10.1155/crit/3745524

**Published:** 2026-10-31

**Authors:** Tomota Okabe, Yuki Nakajima, Kengo Iwatsuki, Ryoichi Maenosono, Takuya Matsuda, Shuya Tsuchida, Takaki Hanamori, Yuya Fujiwara, Keita Nakamori, Tomohisa Matsunaga, Yuki Yoshikawa, Takuya Tsujino, Hideki Matsumura, Hayahito Nomi, Akira Ashida, Haruhito Azuma

**Affiliations:** ^1^ Department of Urology Osaka Medical and Pharmaceutical University Takatsuki Osaka Japan ompu.ac.jp; ^2^ Department of Urology Moriguchi Keijinkai Hospital Moriguchi Osaka Japan dlut.edu.cn; ^3^ Blood Purification Center Osaka Medical and Pharmaceutical University Hospital Takatsuki Osaka Japan; ^4^ Department of Pediatrics Osaka Medical and Pharmaceutical University Takatsuki Osaka Japan ompu.ac.jp; ^5^ Department of Urology Osaka Medical and Pharmaceutical University Mishima-Minami Hospital Takatsuki Osaka Japan dlut.edu.cn

## Abstract

Nail‐patella syndrome (NPS) is a rare disease characterized by fingernail dysplasia, patellar dysplasia, elbow deformities, and iliac horns, with a prevalence of 1 in 50,000 children. Most cases are caused by *LMX1B* mutations. Approximately 10%–40% of patients with NPS develop renal dysfunction in early childhood, whereas approximately 30% progress to end‐stage renal disease (ESRD). In addition, a few reports describe NPS without *LMX1B* mutations, but the mechanism affecting renal function is still unclear. In particular, nail‐patella‐like renal disease (NPLRD) presents solely with nephropathy without the physical changes that are characteristic of NPS. Herein, we report a case of clinicopathological NPLRD with characteristic NPS findings on electron microscopy but without physical features or *LMX1B* mutation, as confirmed by whole exome sequencing. The patient subsequently progressed to ESRD and underwent living‐related kidney transplantation. Seven years posttransplantation, no renal function‐related events occurred, and the patient returned to social activities without complications.

## 1. Introduction

Nail‐patella syndrome (NPS) is an autosomal dominant genetic disorder characterized by a tetralogy of fingernail, patellar, elbow, and iliac horn dysplasia, which can also cause eye disease and nephropathy. NPS is thought to be caused by a heterozygous mutation in *LMX1B*, which encodes LMX1B, a LIM homeodomain transcription factor located on chromosome 9q33.3 [1]. LMX1B is expressed in the dorsal mesenchyme of the developing vertebrate limbs and plays an important role in the dorsoventral axis formation. It regulates the development of dorsal distal end structures, such as the claw and patella, by activating dorsalization gene expression and repressing the expression of ventralization genes. Additionally, it regulates brain, spinal cord, eye, ear, kidney, and limb development [2–4]. The clinical phenotype associated with *LMX1B* abnormalities is highly variable, and a clear genotype–phenotype correlation has not been fully established. In addition to classical NPS, some patients predominantly or exclusively develop renal manifestations without the characteristic nail and skeletal abnormalities. This phenotype has been described as NPLRD.

Approximately 40% of patients with NPS develop nephropathy, ranging from mild proteinuria to end‐stage renal disease (ESRD) [5]. Histologically, light microscopy (LM) reveals various nonspecific findings, from mild glomerular abnormalities to focal segmental glomerulosclerosis. However, electron microscopy (EM) shows specific NPS‐related features, including irregular thickening of the glomerular basement membrane (GBM) with Type III collagen deposition, electron‐lucent areas (moth‐eaten appearance, MEA), and partial loss of podocyte foot processes [5–7].

By contrast, NPLRD is characterized by renal dysfunction similar to NPS but without its physical features. The phenotype of *LMX1B*‐related disease is variable, and renal involvement may occur predominantly or in isolation without typical nail or skeletal abnormalities. Several reports have described *LMX1B* mutations similar to those in NPS [5, 7], supporting a phenotypic continuum between NPS and NPLRD, although the exact cause remains unclear.

Herein, we report a case of a patient clinicopathologically diagnosed with NPLRD based on histological findings characteristic of NPS but without genetic abnormalities. The patient received a living‐related kidney transplant. Seven years after transplantation, renal function remained stable, and the patient resumed social activity without the need for hemodialysis.

## 2. Case Presentation

A 22‐year‐old female with ESRD due to NPLRD underwent a living‐related kidney transplantation from 50‐year‐old mother in Osaka Medical and Pharmaceutical University in 2019. In 2001, proteinuria was first detected at the age of 4 following an episode of acute pharyngitis (Table 1). At the time, this patient had proteinuria without hematuria or systemic edema. The patient was followed without pharmaceutical treatment. Proteinuria persisted during the subsequent 2 years; a renal biopsy was performed at the age of 6 years; however, no significant pathological findings were identified by either LM or EM. Dipyridamole therapy was initiated after the first biopsy. However, proteinuria subsequently worsened, and a second renal biopsy was performed at the age of 10 years. LM showed minor glomerular changes, mild tubular atrophy, and interstitial fibrosis, whereas EM revealed diffuse thickening of the GBM, partially electron‐lucent areas (MEA), and podocyte foot process effacement—findings characteristic of NPS (Figure 1). Physical examination revealed a mild limitation in elbow extension but no other NPS‐specific features. Based on these findings, the pediatric team clinicopathologically diagnosed the patient with NPLRD. Genetic analysis of leukocyte DNA from peripheral blood using the Sanger method did not identify any *LMX1B* mutations.

**Table 1:** The clinical course in this case.

Year	Age	Clinical course	Key findings/treatment
2001	4	First detection of proteinuria	Proteinuria was detected following an episode of acute pharyngitis (no hematuria or systemic edema). Following without pharmacological treatment.
2003	6	First kidney biopsy	First kidney biopsy because of persistent proteinuria: No significant pathological abnormalities were identified by LM or EM. Dipyridamole therapy was initiated.
2007	10	Second kidney biopsy and diagnosis of NPLRD	Second kidney biopsy because of worsening proteinuria: EM showed characteristic of NPS‐related renal disease. The clinicopathological diagnosis of NPLRD (no *LMX1B* mutation).
2013	16	Progression to nephrotic syndrome	Despite treatment, proteinuria and renal dysfunction progressed. The patient was hospitalized with nephrotic syndrome.
2016	19	Referral for renal replacement therapy	The patient was referred to our department for renal replacement therapy: WES of the recipient and potential parents did not identify pathogenic or clearly causative *LMX1B* variants.
2016–2019	19–22	Hemodialysis	The patient received hemodialysis for approximately three years while considering kidney transplantation.
2019	22	Kidney transplantation	The patient underwent living‐related kidney transplantation from her mother. No evidence of acute rejection was observed.
2020	22–23	Modification of immunosuppression	Six months after transplantation, tacrolimus was reduced and everolimus was added, without rejection.
2019‐2026	22–29	Posttransplant follow‐up	Two episodes of fever and dehydration were associated with transient increases in serum creatinine and proteinuria.
2026	29	Transplanted kidney biopsy and states	No evidence of recurrent disease or rejection was identified. Graft function remained stable, with serum creatinine of 0.8–0.9 mg/dL.

Abbreviations: EM, electron microscopy; LM, light microscopy; WES, whole exome sequencing.

**Figure 1:**
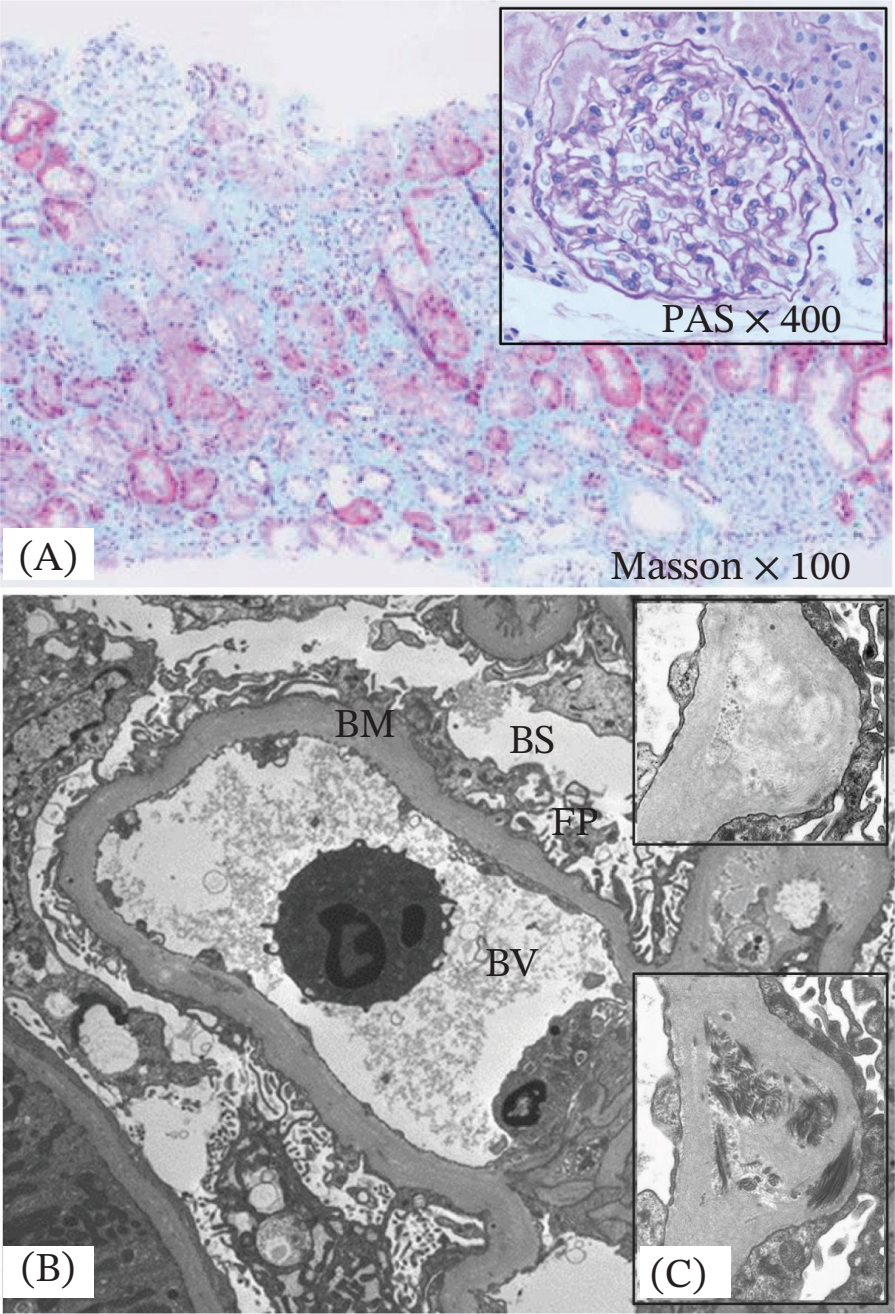
Kidney biopsy at the age of 10 years. (A) PAS and Masson′s trichrome stains showing mild glomerular changes and interstitial fibrosis. (B) Electron microscopy showing diffuse thickening of the glomerular basement membrane with partially electron‐lucent areas (moth‐eaten appearance). (C) Tannic acid staining revealing scattered collagen fibrils throughout full thickness of the basement membrane, with focal nodular protrusions towards the epithelial side. BM, basement membrane; BS, Bowman′s space; BV, blood vessel; FP, foot process; PAS, periodic acid‐Schiff.

Despite treatment with angiotensin converting enzyme inhibitors/angiotensin receptor blockers, the patient was hospitalized at the age of 16 years with nephrotic syndrome due to worsening proteinuria and renal function. At the age of 19 years, the patient was referred to our department for renal replacement therapy. Parents were considered as living kidney donors, and next‐generation whole‐exome sequencing showed no *LMX1B* abnormalities both in the donors and recipient. The patient initially received hemodialysis for 3 years before finally deciding to receive a living donor kidney transplant at the age of 22 years.

The donor was a 50‐year‐old woman with no medical history about renal disease (serum creatinine 0.65 mg/dL, with urine no hematuria or proteinuria; no clinical findings suggestive of hereditary nephropathy were identified). The recipient′s blood type was A, and the donor blood type was O. The human leukocyte antigen (HLA) mismatch was one in HLA‐DR, and the crossmatch examination was negative. Immunosuppression was initiated with tacrolimus, mycophenolate mofetil, methylprednisolone, and basiliximab. Renal blood flow was regularly monitored perioperatively, with no signs of acute rejection. The patient was discharged 18 days after transplantation with decreased serum creatinine (nadir serum creatinine 0.67 mg/dL) and increased urine output. Six months later, tacrolimus was reduced, and everolimus was added. During follow‐up, the patient experienced two episodes of fever and dehydration, which were accompanied by transient increases in serum creatinine and proteinuria. However, both episodes were resolved promptly with appropriate management (Figure 2). A protocol kidney biopsy performed 7 years after transplantation showed no evidence of recurrent disease or rejection (Table 2), and graft function has remained stable (serum creatinine 0.8–0.9 mg/dL). The donor has remained clinically stable to date, with no hematuria, proteinuria, or decline in renal function.

**Figure 2:**
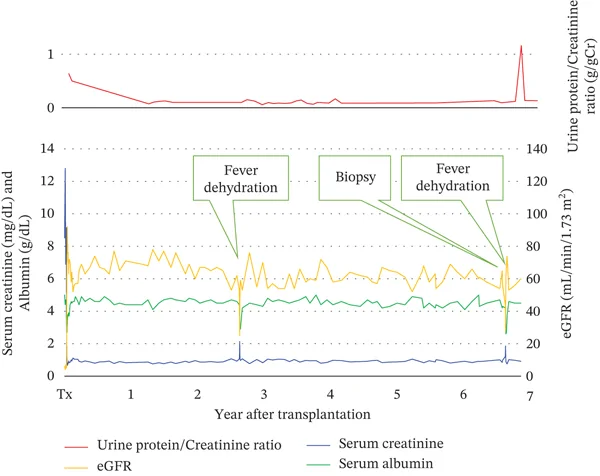
Longitudinal course of renal function following kidney transplantation. Transient deterioration in renal function occurred at approximately 2.5 and 6.5 years after transplantation, likely because of dehydration, but renal function promptly recovered without specific treatment.

**Table 2:** Results of 7 years protocol biopsy after kidney transplantation.

Light microscopy	Immunofluorescence	Electron microscopy
Banff criteria (2019) ● i0, t0, v0, g0, ptc0, C4d0, ci1, ct1, cv1, cg0, mm0, aah0, ti1, i‐IFTA2, t‐IFTA0, pvl0 ● SV40 staining (−)	Glomerular ● IgG (±) ● IgA (−) ● IgM (weak+) ● C3 (−) ● C1q (−) ● Control (−)	● No electron‐dense deposits. ● No moth‐eaten changes or disruption of the glomerular basement membrane. ● Focal foot process effacement with mild podocyte swelling.

## 3. Discussion

NPS and NPLRD are rare causes of renal dysfunction in childhood, with 10%–40% of affected children developing albuminuria, proteinuria, and often microscopic hematuria. Diagnosis is often difficult owing to the lack of specific histologic features on LM. However, EM reveals disease‐specific findings, such as GBM thickening and MEA [6, 8]. Early intervention is necessary to prevent progression to renal failure in childhood.

NPS is predominantly caused by heterozygous pathogenic variants in *LMX1B* [9]. The *LMX1B* comprises eight exons and encodes a transcription factor containing two LIM domains and one homeodomain [10]. Numerous pathogenic variants have been reported in association with NPS [5], with haploinsufficiency of *LMX1B* considered the major pathogenic mechanism. However, a proportion of clinically diagnosed NPS cases lack identifiable pathogenic variants in the coding regions of the gene [9, 11]. Functional studies have also shown that specific missense variants, such as R246Q, can reduce the transcriptional activity of *LMX1B* [7]. Nevertheless, the genotype–phenotype relationship of *LMX1B*‐associated disease remains incompletely understood.

In the present case, no pathogenic *LMX1B* variant was identified. Nevertheless, the diagnosis of NPLRD was supported by the characteristic renal pathological findings. EM demonstrated diffuse irregular thickening of GBM with MEA, which represents a characteristic ultrastructural feature of NPS/NPLRD. Furthermore, tannic acid staining revealed transversely striated collagen fibrils scattered throughout the full thickness of the GBM, consistent with the characteristic intramembranous Type III collagen deposition described in NPS/NPLRD. COL4A3‐, COL4A4‐, and COL4A5‐related nephropathies, including atypical Alport syndrome, were considered; however, preserved Type IV collagen expression and the absence of characteristic GBM splitting, lamellation, and basket‐weave appearance did not support these diagnoses [12]. Hereditary podocytopathies were also considered, but the prominent intrinsic GBM abnormalities and collagen fibrils could not be explained by primary podocytopathy alone. Collagenofibrotic glomerulopathy was also considered in the differential diagnosis; however, Type III collagen fibrils are predominantly deposited in the mesangial and subendothelial areas, whereas in NPS/NPLRD, they are characteristically distributed within the GBM [13]. The combination of irregular GBM thickening, MEA, and intramembranous collagen fibrils provided important pathological support for the diagnosis of NPLRD.

Our patient showed declining renal function, which was clinicopathologically diagnosed as NPLRD without obvious genetic mutations. The patient had undergone conservative treatment during childhood; however, the patient′s renal failure progressed. Eventually, the patient′s mother was selected as the living donor for a kidney transplant. Few reports exist on living‐donor kidney transplantation for NPD/NPLRD. Chan et al. [14] performed a living‐donor kidney transplantation in an NPS patient, reporting no recurrence of renal failure after 18 months. Similarly, Nair et al. [15] described a 29‐year‐old man diagnosed with NPS who underwent living‐donor kidney transplantation from his 46‐year‐old mother, with normal renal function 4 years following surgery. No genetic mutations were found in either parent; however, the patient had a missense mutation at codon 725, where thymine was replaced with cytosine [15].

Recurrence following kidney transplantation in hereditary nephrotic syndrome is rare. Bierzynska et al. [16] reported that kidney transplantation has been performed in nephrotic syndrome cases with monogenic disease, with posttransplant recurrence rates of 0%. However, few reports have described the detailed course of renal transplantation in patients with NPS and NPLRD. A limitation is that no gene abnormalities were found in the screening of the patient and parents, making it difficult to confirm a diagnosis of NPLRD. However, WES has inherent limitations and may fail to detect deep intronic or regulatory variants, structural variants, and copy‐number alterations. Therefore, an as‐yet unidentified genetic alteration, including an undetected *LMX1B* variant, cannot be excluded. However, the morphological features, such as MEA of the GBM, were consistent with NPLRD and distinct from Alport syndrome. Additional limitations include incomplete technical details of the original WES analysis and the lack of quantitative assessment of urinary protein excretion in the donor. However, qualitative urinalysis before transplantation showed no evidence of proteinuria.

In conclusion, we report a case of kidney transplantation in a patient with ESRD due to clinicopathological NPLRD, without an identifiable pathogenic genetic mutation. The patient maintained stable graft function over 7 years, with no recurrence of proteinuria. To the best of our knowledge, this is the first report describing long‐term posttransplant clinical outcomes together with protocol allograft biopsy findings in a patient with clinicopathological NPLRD. Considering the patient′s young age, we believe that kidney transplantation may represent a feasible and effective treatment for NPLRD‐associated ESRD. Further studies are warranted to clarify long‐term outcomes and the risk of disease recurrence in this rare disease.

**Nomenclature**
EM: electron microscopy
ESRD: end‐stage renal disease
FSGS: focal segmental glomerulosclerosis
GBM: glomerular basement membrane
LM: light microscopy
MEA: moth‐eaten appearance
NPS: nail‐patella syndrome
NPLRD: nail‐patella‐like renal disease

## Author Contributions

T.O., T.M., S.T., and Y.N. analyzed the data and wrote the manuscript. T.H., Y.F., K.N., and T.M. collected and analyzed the data. Y.Y., T.T., H.N., H.M., A.A., and H.A. helped with manuscript preparation. R.M. designed, supervised, wrote, and edited the manuscript. T.O., Y.N., and K.I. have contributed equally to this work.

## Funding

No funding was received for this manuscript.

## Consent

Written informed consents about publishment were taken from the donor and recipient prior to submission of the manuscript.

## Conflicts of Interest

The authors declare no conflicts of interest.

## Data Availability

The data supporting the findings of this study are available upon request from the corresponding author (R.M.).
